# CAR-T cell therapy for solid tumors: antigen landscape, biological barriers, and engineering strategies for clinical translation

**DOI:** 10.3389/fimmu.2026.1968080

**Published:** 2026-10-09

**Authors:** Qian Niu, Meng Li, Cheng-Yi Zhang, Qian Li, Meng Wang, An-Dong Liu

**Affiliations:** 1 Department of Respiratory and Critical Care Medicine, Third Hospital of Shanxi Medical University, Shanxi Bethune Hospital, Shanxi Academy of Medical Sciences, Tongji Shanxi Hospital, Taiyuan, China; 2 Institute of Hematology, Union Hospital, Tongji Medical College, Huazhong University of Science and Technology, Wuhan, China; 3 School of Basic Medicine, Tongji Medical College, Huazhong University of Science and Technology, Wuhan, Hubei, China; 4 Department of Pathology, Zhongnan Hospital of Wuhan University, Wuhan, China

**Keywords:** car-t, immunotherapy, solid tumors, therapeutic strategies, tumor antigens

## Abstract

Chimeric Antigen Receptor T (CAR-T) cell therapy has emerged as a promising therapeutic option, particularly for refractory and relapsed leukemias. However, its efficacy in treating solid tumors, including lung, ovarian, gastric, and breast cancers, remains limited by several challenges. Recent studies have highlighted CAR-T cell immunotherapy as a potential treatment for these malignancies, utilizing genetically engineered T cells that target specific tumor antigens. For successful therapy, it is crucial to identify antigens that are selectively expressed on tumor cells but not on normal tissues. In lung cancer, however, the lack of specific tumor-associated antigens, the immunosuppressive tumor microenvironment, limited CAR-T cell infiltration, and off-target effects complicate the development of effective CAR-T therapies. This review outlines the fundamental structure and generation of CAR-T cells, summarizes key tumor-associated antigens targeted in clinical trials for these cancers, and discusses the current challenges and emerging strategies. The goal is to provide insights and potential approaches to enhance the design and execution of preclinical and clinical CAR-T cell therapies for these solid tumors.

## 1 Introduction

Despite major advances in surgery, radiotherapy, chemotherapy, targeted therapy, and immune checkpoint blockade, solid tumors remain a leading cause of cancer-related mortality worldwide (1). Although these treatment modalities have improved survival in selected patient populations, durable clinical responses remain limited by tumor heterogeneity, therapeutic resistance, and immune evasion (2). Consequently, the development of innovative therapeutic strategies capable of eliciting durable and tumor-specific immune responses has become a major focus of contemporary cancer research.

Adoptive cell therapy has emerged as one of the most transformative approaches in cancer immunotherapy. Among these strategies, chimeric antigen receptor T (CAR-T) cell therapy has achieved unprecedented clinical success in hematological malignancies, particularly B-cell acute lymphoblastic leukemia and diffuse large B-cell lymphoma, leading to multiple regulatory approvals and establishing engineered T-cell therapy as a new therapeutic paradigm (3). Unlike conventional T-cell receptor (TCR)-based therapies, CAR-T cells recognize cell-surface antigens in a major histocompatibility complex (MHC)-independent manner, enabling potent and direct cytotoxic activity against malignant cells.

Despite these remarkable achievements, the clinical translation of CAR-T cell therapy to solid tumors has been considerably more challenging. Several biological barriers collectively limit therapeutic efficacy, including the scarcity of truly tumor-specific antigens, substantial inter- and intratumoral heterogeneity, antigen-loss-mediated immune escape, inadequate trafficking and infiltration into tumor tissues, limited *in vivo* persistence, and the establishment of a highly immunosuppressive tumor microenvironment (TME) (4–6). In addition, many target antigens expressed by solid tumors are also found at low levels in normal tissues, creating the risk of on-target/off-tumor toxicity and restricting the therapeutic window of CAR-T cell therapies (7). These challenges highlight the fundamental biological differences between hematological malignancies and solid tumors and underscore the need for next-generation engineering strategies.

Recent advances in synthetic biology, genome editing, single-cell sequencing, and tumor immunology have accelerated the development of novel CAR-T platforms designed to overcome these limitations. Emerging approaches, including multi-antigen targeting, armored CAR-T cells, cytokine-secreting CAR constructs, chemokine receptor engineering, immune checkpoint-resistant CAR-T cells, and rational combination therapies, have demonstrated encouraging preclinical and early clinical activity against solid tumors (8). Collectively, these innovations are reshaping the landscape of adoptive cellular immunotherapy and expanding the therapeutic potential of CAR-T cells beyond hematological cancers. Existing reviews have typically focused on CAR structure, target antigens, or engineering strategies separately. However, a comprehensive evaluation integrating antigen landscape, biological barriers, engineering solutions, and their clinical maturity remains lacking.

In this review, we provide an integrated and translational perspective on CAR-T cell therapy for solid tumors. Unlike many previous reviews that focus primarily on CAR design, target antigens, or engineering strategies individually, we link three interconnected dimensions of solid-tumor CAR-T therapy: antigen selection, biological barriers, and engineering solutions. We critically evaluate the clinical maturity of major CAR-T platforms, distinguish approaches supported by clinical evidence from those remaining at the preclinical stage, and highlight key unresolved challenges that continue to limit durable responses. Particular emphasis is placed on emerging concepts including multi-antigen targeting, armored CAR-T cells, *in vivo* CAR-T generation, and strategies designed to overcome T-cell exhaustion and tumor microenvironment-mediated immunosuppression. By integrating mechanistic insights with available clinical evidence, this review aims to provide a translational framework for understanding current progress, identifying key bottlenecks, and guiding future development of CAR-T therapies for solid malignancies.

## 2 Evolution of CAR design: from first-generation constructs to next-generation engineered CAR-T cells

Chimeric antigen receptors (CARs) are synthetic receptors that redirect T cells toward predefined tumor-associated antigens (TAAs) in a major histocompatibility complex (MHC)-independent manner. A canonical CAR consists of an extracellular antigen-recognition domain, a hinge/spacer region, a transmembrane domain, and an intracellular signaling module that collectively determine antigen recognition, signal transduction, and T-cell activation (**Figures 1A, B**).

**Figure 1:**
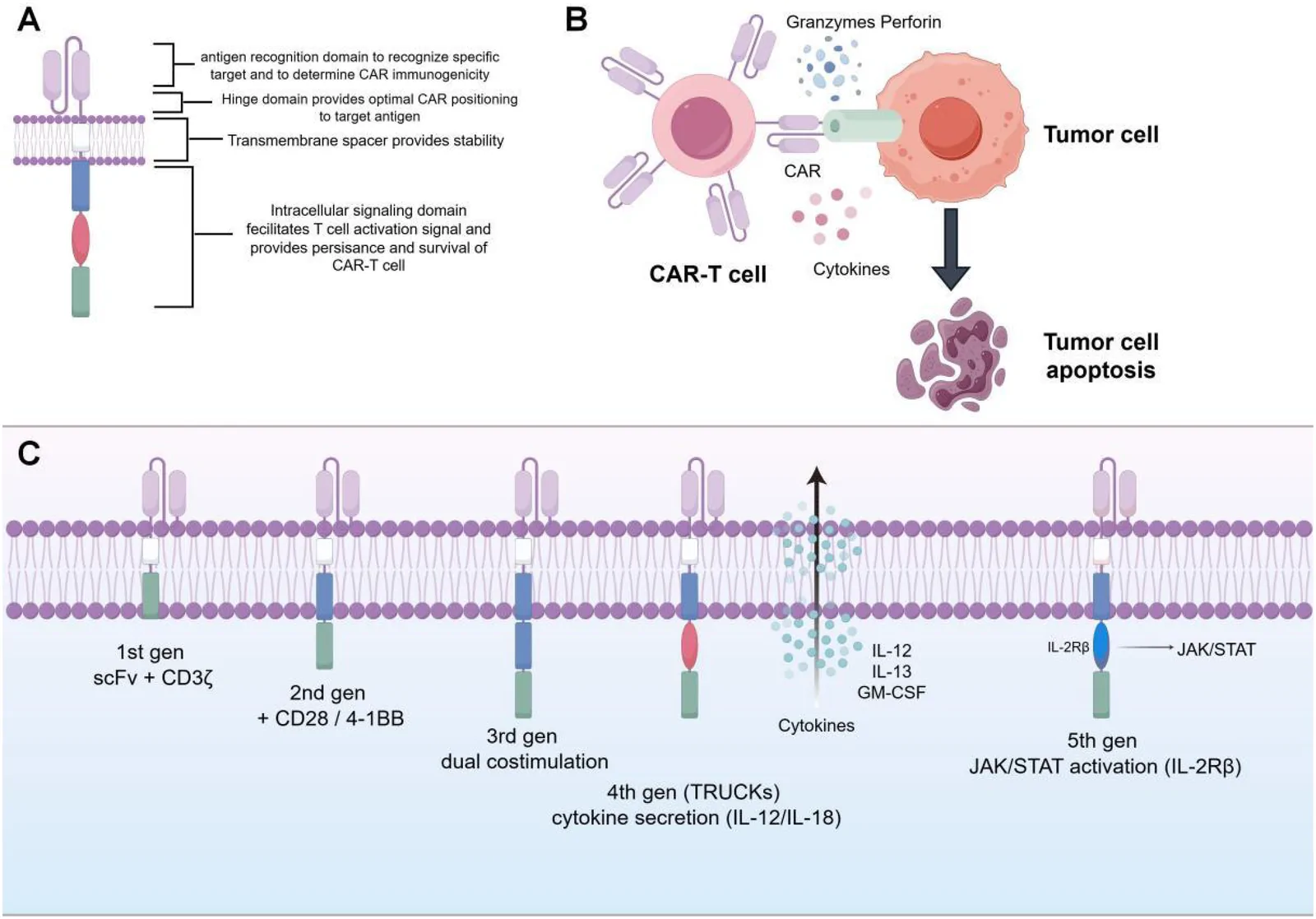
Structure, evolution, and antitumor mechanisms of CAR-T cells. **(A)** Structural components of a chimeric antigen receptor, including the antigen-binding domain, hinge region, transmembrane domain, and intracellular signaling domain. **(B)** CAR-T-cell-mediated tumor killing through antigen recognition, cytokine secretion, perforin/granzyme release, and induction of tumor-cell apoptosis. **(C)** Evolution of CAR designs from first- to fifth-generation constructs, highlighting the incorporation of costimulatory domains, cytokine-inducible modules, and JAK/STAT signaling elements to enhance proliferation, persistence, and antitumor activity.

The evolution of CAR design has largely been driven by efforts to improve T-cell persistence, functionality, and antitumor activity (9) (**Figure 1C**). First-generation CARs contained only the CD3ζ signaling domain and were capable of mediating antigen-specific cytotoxicity; however, they exhibited limited proliferation, poor persistence, and insufficient clinical efficacy (10). To address these limitations, second-generation CARs incorporated a single costimulatory domain, most commonly CD28 or 4-1BB, which significantly enhanced T-cell expansion, cytokine production, and *in vivo* persistence (11, 12). These constructs form the foundation of most currently approved CAR-T cell products. Notably, CD28-based CARs generally promote rapid effector responses, whereas 4-1BB-containing CARs are associated with improved persistence and memory formation.

Third-generation CARs further integrated two costimulatory domains, such as CD28 and 4-1BB, OX40, ICOS, or CD27, to enhance signaling strength and T-cell activation (13, 14). Although these designs demonstrated improved functionality in preclinical studies, their clinical superiority over second-generation CARs remains uncertain, suggesting that enhanced intracellular signaling alone may be insufficient to overcome the challenges encountered in solid tumors.

Subsequent advances in synthetic biology led to the development of fourth-generation CARs, also known as T cells redirected for universal cytokine-mediated killing (TRUCKs). These engineered cells incorporate inducible cytokine-expression systems that enable the local release of immune-stimulatory molecules, including IL-12 and IL-18, following antigen recognition (15). By recruiting innate immune cells and enhancing antigen presentation, TRUCKs partially remodel the immunosuppressive tumor microenvironment. Fifth-generation CARs further integrate cytokine receptor signaling domains, such as truncated IL-2Rβ motifs, enabling activation of JAK–STAT pathways in parallel with conventional CAR signaling and thereby enhancing proliferative capacity and persistence (8, 16).

Despite increasingly sophisticated receptor architectures, the clinical efficacy of CAR-T therapy in solid tumors remains limited. Improvements in activation and persistence have not fully overcome major barriers such as antigen heterogeneity, inadequate tumor infiltration, T-cell exhaustion, and tumor microenvironment-mediated immunosuppression (4–6). For example, while 4-1BB signaling enhances persistence, it does not substantially improve tumor trafficking, whereas cytokine-secreting CARs may introduce additional safety concerns related to excessive immune activation. Similarly, although fifth-generation CARs have demonstrated encouraging preclinical activity, clinical evidence supporting their superiority remains limited.

Collectively, the evolution of CAR design reflects a transition from enhancing antigen-specific cytotoxicity toward comprehensive cellular reprogramming. Nevertheless, no single CAR architecture has fully resolved the complex biological barriers presented by solid tumors. Future progress will likely depend on the integration of optimized CAR constructs with strategies targeting antigen heterogeneity, immune suppression, and tumor-specific trafficking, as discussed in the following sections.

Although substantial advances in CAR architecture have significantly improved T-cell activation, persistence, and antitumor activity, the therapeutic efficacy of CAR-T cells ultimately depends on a complex multistep process that extends beyond receptor design (1, 17). Following leukapheresis, genetic modification, and ex vivo expansion, CAR-T cells must successfully traffic to tumor sites, infiltrate tumor tissues, recognize tumor-associated antigens, and maintain sustained effector function within an immunosuppressive tumor microenvironment (18, 19). Failure at any of these stages can markedly compromise therapeutic efficacy, particularly in solid tumors where physical barriers, antigen heterogeneity, and immune suppression frequently limit CAR-T-cell activity.

Upon antigen recognition, CAR-T cells eliminate tumor cells through multiple mechanisms, including perforin/granzyme-mediated cytotoxicity, Fas/FasL-induced apoptosis, and the secretion of cytokines that modulate the tumor microenvironment and recruit endogenous immune cells. These complementary effector functions may also contribute to the elimination of antigen-negative tumor cells through indirect bystander effects. The manufacturing workflow of CAR-T-cell products and the major mechanisms underlying CAR-T-cell-mediated tumor eradication are illustrated in **Figure 2**.

**Figure 2:**
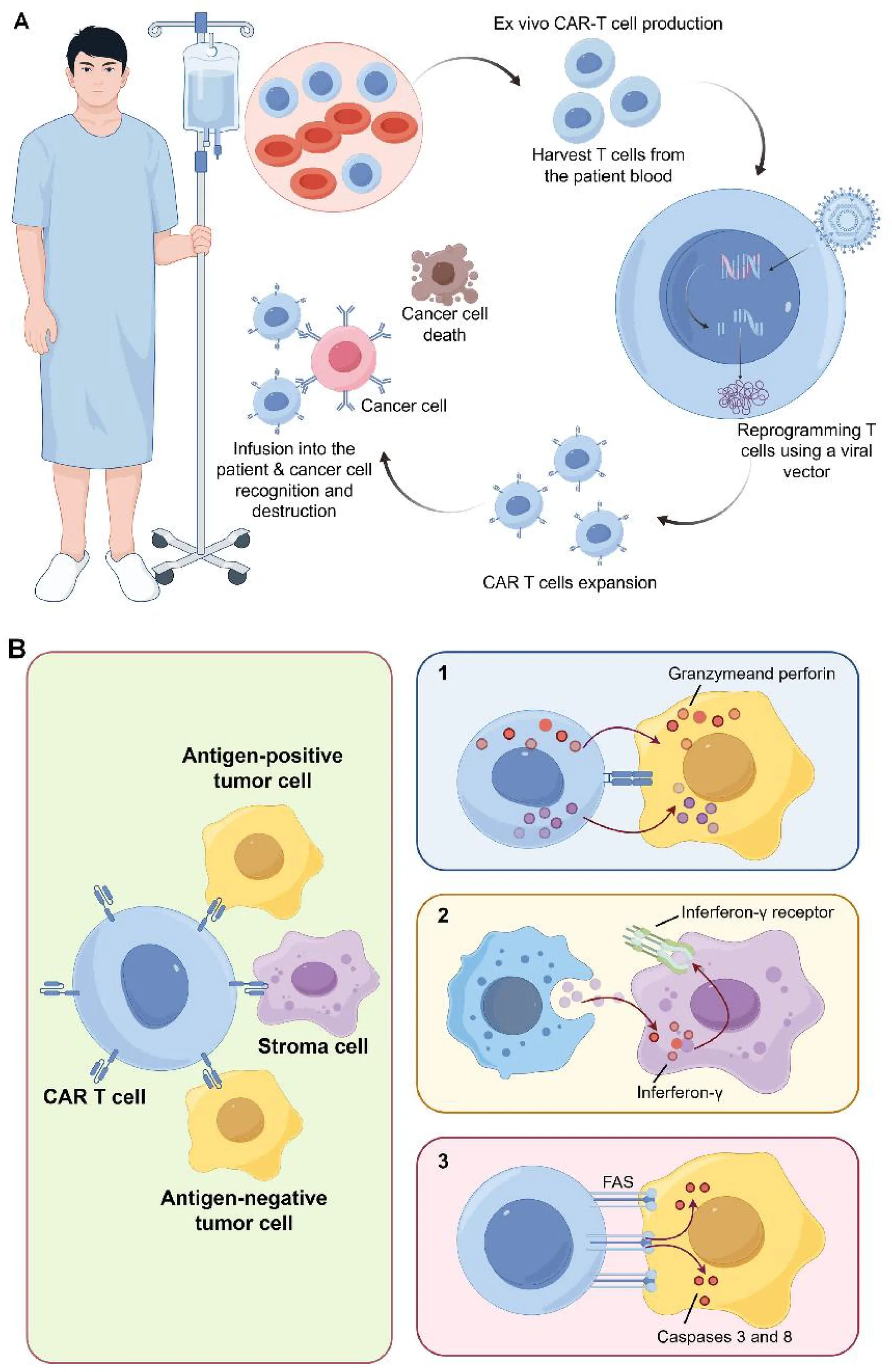
Overview of CAR-T-cell therapy and mechanisms of tumor elimination in solid tumors. **(A)** Ex vivo manufacturing process of CAR-T cells, including leukapheresis, T-cell isolation, genetic modification, expansion, and adoptive transfer. Following infusion, CAR-T cells traffic to tumor sites and recognize tumor-associated antigens. **(B)** Major mechanisms of CAR-T-cell–mediated antitumor activity. CAR-T cells directly kill antigen-positive tumor cells through perforin/granzyme release, remodel the tumor microenvironment through cytokine secretion (e.g., IFN-γ), and induce Fas/FasL-dependent apoptosis that may contribute to bystander elimination of antigen-negative tumor cells. These complementary mechanisms collectively enhance antitumor efficacy and help limit immune escape.

## 3 Antigen landscape for CAR-T therapy in solid tumors

The selection of an appropriate target antigen is a prerequisite for successful CAR-T cell therapy. An ideal target should be highly and uniformly expressed on tumor cells while exhibiting minimal expression in normal tissues. In hematological malignancies, lineage-restricted antigens such as CD19 largely fulfill these requirements, contributing to the remarkable clinical success of CAR-T therapy (20). In contrast, identifying suitable targets in solid tumors remains considerably more challenging because most candidate antigens display heterogeneous expression patterns, incomplete tumor specificity, and dynamic changes during disease progression (21, 22).

To provide an overview of the current antigen landscape, the major CAR-T targets under investigation for solid tumors are summarized in **Table 1**. These antigens differ substantially in tumor specificity, biological function, and therapeutic limitations, highlighting the challenges associated with balancing tumor coverage and safety. Representative preclinical and clinical evidence supporting these targets and engineering strategies is summarized separately in **Table 2**.

**Table 1:** Major CAR-T cell targets in solid tumors.

Antigen	Tumor types	Advantages	Major limitations	Clinical status
EGFR	NSCLC, HNSCC, Glioblastoma	High expression	Expression in normal epithelium; risk of on-target/off-tumor toxicity	Phase I
HER2	Gastric cancer, Breast cancer, Ovarian cancer	Strong oncogenic dependence	Severe toxicity reported in early CAR-T studies; heterogeneous expression	Phase I
c-MET	Breast cancer, NSCLC	Associated with aggressive disease and poor prognosis	Variable expression among patients	Phase I completed
AXL	Triple-negative breast cancer, NSCLC	Associated with metastasis and therapy resistance	Expression in some normal tissues	Phase I
ROR1	Lung, Breast, Ovarian, Pancreatic cancer	Limited adult tissue expression; functionally important for tumor survival	Low-level normal tissue expression raises safety concerns	Phase I
CLDN18.2	Gastric cancer, Gastroesophageal cancer	Highly tumor-restricted; favorable safety profile	Restricted to specific tumor subtypes	Phase I/II
Mesothelin (MSLN)	Mesothelioma, Ovarian, Lung, Pancreatic cancer	Broad expression across solid tumors; attractive pan-cancer target	Antigen shedding and heterogeneous expression	Multiple Phase I/II
CEA	NSCLC, Gastric, Colorectal cancer	Highly expressed in epithelial malignancies	Low-level expression in normal gastrointestinal tissues	Multiple Phase I
MUC1	Gastric, Pancreatic, Breast cancer	Widely expressed and associated with tumor progression	Broad expression in normal epithelium	Early clinical evaluation
MUC16 (CA125)	Ovarian cancer	Highly expressed in ovarian cancer; mediates metastasis	Antigen shedding may impair targeting	Phase I
DLL3	Small-cell lung cancer (SCLC)	Minimal expression in normal tissues; highly specific for neuroendocrine tumors	Restricted to neuroendocrine malignancies	Phase I
CSPG4	Triple-negative breast cancer, Melanoma	Associated with aggressive tumor phenotype	Expression heterogeneity	Phase I/II
CD70	Ovarian cancer, RCC, Glioma	Limited normal tissue distribution	Potential immune-related toxicity	Phase I
FRα	Triple-negative breast cancer, Ovarian cancer	Highly expressed in aggressive tumors	Variable expression levels	Phase II completed
AMHR2	Ovarian cancer, Endometrial cancer	Highly restricted normal tissue expression	Limited clinical experience	Phase I completed

**Table 2:** Representative preclinical and clinical evidence supporting CAR-T cell therapy in solid tumors.

Strategy / target	Tumor type	Study type	Sample size	Key efficacy findings	Major safety findings	Current status	Ref.
CLDN18.2 CAR-T (CT041)	Gastric / gastroesophageal cancer	Phase I	n=37	ORR 48.6%, DCR 73.0%; ORR 57.1% in gastric cancer	Mainly grade 1–2 CRS; no grade ≥3 CRS or neurotoxicity	Most advanced CAR-T program in GI tumors	(38)
Mesothelin CAR-T + Pembrolizumab	Malignant pleural mesothelioma	Phase I	n=27	Median OS 23.9 months; durable disease control; complete metabolic responses observed	Well tolerated; no unexpected severe toxicity	Proof-of-concept for CAR-T + checkpoint blockade	(75)
EGFR/IL13Rα2 Dual-target CAR-T	Recurrent glioblastoma	Phase I	n=18	Objective tumor regression and prolonged disease control in some patients	Manageable neurotoxicity	Early clinical validation of dual-target CARs	(76)
PSMA-DN TGFβR CAR-T	Metastatic castration-resistant prostate cancer	Phase I	n=13	PSA decline and disease stabilization in subsets of patients	CRS manageable; one treatment-related death reported	First armored CAR-T clinical evidence	(77)
CLDN6 CAR-T + CARVac (RNA vaccine)	Testicular cancer and other CLDN6-positive tumors	Phase I	n=22	Durable responses including complete responses	Acceptable safety profile	First CAR-T/RNA-vaccine combination	(78)
NR4A-deficient CAR-T	Melanoma / colon cancer models	Preclinical	Mouse models	Enhanced persistence, cytokine production, and tumor control	Preclinical safety acceptable	Exhaustion-resistance strategy	(67)
HER2/IL13Rα2/EphA2 Trivalent CAR-T	Glioblastoma	Preclinical	Mouse models	Superior tumor coverage and control compared with single-target CAR-T cells	Not fully evaluated	Multi-antigen targeting strategy	(79)
TanCAR (HER2 + IL13Rα2)	Glioblastoma	Preclinical	Mouse models	Reduced antigen escape and improved antitumor efficacy	Preclinical only	Dual-antigen recognition platform	(80)
FRβ-targeted CAR-T against TAMs	Ovarian cancer	Preclinical	Mouse models	Reprogrammed immunosuppressive TME and enhanced endogenous immunity	Not yet clinically validated	TAM-targeting strategy	(81)
IL-12 / IL-18 Armored CAR-T	Multiple solid tumor models	Preclinical	Mouse models	Improved infiltration, persistence, and antitumor activity	Potential cytokine-associated toxicity	Cytokine-armored CAR-T platform	(82)

### 3.1 Receptor tyrosine kinases: broad applicability but limited specificity

Receptor tyrosine kinases (RTKs) represent one of the most extensively studied classes of CAR-T targets in solid tumors. EGFR and HER2 are frequently overexpressed in non-small cell lung cancer, glioblastoma, breast cancer, gastric cancer, and other epithelial malignancies (23–28). Preclinical studies have demonstrated potent antitumor activity of EGFR- and HER2-directed CAR-T cells (28–30). However, the fatal pulmonary toxicity reported in an early HER2 CAR-T clinical trial highlighted the risks associated with targeting antigens that are also expressed in normal tissues, underscoring the challenge of achieving an adequate therapeutic window.

Other RTK-related targets, including c-MET, AXL, and ROR1, have attracted increasing attention because of their involvement in tumor invasion, metastasis, epithelial–mesenchymal transition, and therapeutic resistance (31–36). Among these, ROR1 is particularly attractive due to its relatively restricted expression in normal adult tissues and its re-expression in multiple malignancies. Emerging approaches incorporating dual-antigen recognition and synthetic Notch circuits may further improve the specificity of ROR1-targeted CAR-T therapies (35, 36).

Overall, RTK-directed CAR-T therapies offer broad patient applicability but remain limited by on-target/off-tumor toxicity and antigen heterogeneity.

### 3.2 Adhesion and surface molecules: current clinical frontrunners

Compared with RTKs, several adhesion and surface molecules exhibit more restricted expression patterns and may therefore provide improved safety profiles.

Among these targets, CLDN18.2 has emerged as one of the most promising antigens for gastrointestinal malignancies. As a stomach-specific tight-junction protein exposed during malignant transformation, CLDN18.2 demonstrates high tumor selectivity and potent antitumor activity in preclinical studies (37, 38). The clinical success of CLDN18.2-targeted antibody therapies further supports its translational potential.

Mesothelin (MSLN) is another clinically advanced target that is highly expressed in mesothelioma, pancreatic cancer, ovarian cancer, and lung adenocarcinoma (39–41). Owing to its broad distribution across multiple solid tumors, mesothelin-directed CAR-T cells are currently being evaluated in numerous clinical trials and combination strategies designed to overcome tumor microenvironment-mediated resistance.

Current clinical experience suggests that CLDN18.2 and mesothelin are among the most advanced CAR-T targets in solid tumors, although durable responses remain limited by tumor heterogeneity and immune suppression.

### 3.3 Oncofetal and emerging highly selective targets

Oncofetal antigens and mucin-associated proteins, including CEA, MUC1, and MUC16, are broadly expressed across multiple epithelial malignancies and have been widely investigated as CAR-T targets (42–49). Their broad applicability provides access to large patient populations; however, residual expression in normal tissues and heterogeneous tumor expression continue to present safety and efficacy challenges.

Recent efforts have increasingly focused on highly selective targets such as DLL3, CD70, CSPG4, FRα, and AMHR2 (50–59). DLL3 is selectively expressed in neuroendocrine tumors, particularly small-cell lung cancer, while FRα and AMHR2 demonstrate relatively restricted expression in ovarian and gynecologic malignancies. These targets may provide wider safety margins but are applicable only to specific patient subsets.

### 3.4 Lessons from current antigen development

Collectively, currently investigated antigens illustrate a fundamental trade-off between tumor coverage and therapeutic specificity. Broadly expressed targets such as EGFR, HER2, mesothelin, and CEA offer wider patient applicability but are associated with increased risks of on-target/off-tumor toxicity. In contrast, highly restricted antigens including CLDN18.2, DLL3, and AMHR2 provide improved selectivity but are limited to narrower patient populations.

Importantly, clinical experience indicates that antigen selection alone is insufficient to determine therapeutic success. Even highly tumor-selective targets may fail to generate durable responses because of antigen heterogeneity, adaptive antigen loss, inadequate T-cell infiltration, and tumor microenvironment-mediated immunosuppression (21, 22). Consequently, future advances will likely depend on the integration of optimal target selection with innovative engineering strategies designed to overcome these biological barriers.

## 4 Biological barriers limiting CAR-T cell therapy in solid tumors

Despite the remarkable success of CAR-T therapy in hematological malignancies, its efficacy in solid tumors remains limited. Unlike blood cancers, solid tumors present multiple interconnected barriers that impair CAR-T-cell trafficking, persistence, and antitumor activity. These obstacles include limited target specificity, tumor heterogeneity, an immunosuppressive tumor microenvironment (TME), T-cell dysfunction, and adaptive immune escape, all of which contribute to treatment failure (6).

### 4.1 Limited target specificity and tumor heterogeneity

The lack of truly tumor-specific antigens represents a fundamental challenge for CAR-T therapy in solid tumors. Most currently targeted antigens, including EGFR, HER2, mesothelin (MSLN), CEA, and MUC1, are also expressed at low levels in normal tissues, creating the risk of on-target/off-tumor toxicity (7). The fatal pulmonary toxicity observed in an early HER2 CAR-T trial remains a landmark example of this limitation (60).

In addition, substantial intertumoral, intratumoral, and temporal heterogeneity frequently results in uneven antigen expression and promotes antigen-loss-mediated resistance (21, 22, 61, 62). Under immune selection pressure, tumor cells may downregulate or completely lose target antigens, allowing antigen-negative clones to escape CAR-T-cell surveillance (21). These observations have stimulated the development of dual-target, tandem CAR, and logic-gated CAR systems designed to improve tumor coverage while enhancing specificity.

### 4.2 Immunosuppressive tumor microenvironment and T-cell dysfunction

Beyond antigen-related challenges, CAR-T cells must function within a highly suppressive TME composed of regulatory T cells (Tregs), myeloid-derived suppressor cells (MDSCs), tumor-associated macrophages (TAMs), cancer-associated fibroblasts, extracellular matrix components, and inhibitory cytokines (1, 63). These factors collectively impair CAR-T-cell activation, proliferation, and cytotoxicity.

The TME also imposes substantial physical and metabolic barriers. Dense extracellular matrix deposition, abnormal vasculature, hypoxia, nutrient deprivation, and elevated interstitial pressure restrict T-cell trafficking and infiltration (2). Consequently, only a fraction of infused CAR-T cells successfully reach tumor tissues.

Persistent antigen stimulation and chronic exposure to suppressive signals further drive T-cell exhaustion, characterized by reduced proliferation, impaired cytokine secretion, diminished cytotoxicity, and increased expression of inhibitory receptors such as PD-1, TIM-3, LAG-3, TIGIT, and CTLA-4 (64–66). Recent studies have identified the NR4A transcription factor family as a central regulator of exhaustion programs, and genetic disruption of NR4A signaling significantly improves CAR-T-cell persistence and antitumor efficacy in preclinical models (67, 68).

### 4.3 Adaptive immune escape

Adaptive immune escape represents a major barrier to durable clinical responses. Under therapeutic pressure, tumor cells undergo genetic, epigenetic, and transcriptional changes that reduce susceptibility to CAR-T-cell-mediated killing (69). Among these mechanisms, antigen loss or downregulation is the most extensively documented.

Clinical evidence for antigen escape has been reported across multiple tumor types. For example, recurrent glioblastomas following IL13Rα2-targeted CAR-T therapy showed markedly reduced antigen expression (70), while relapse after CD19-directed CAR-T therapy has frequently been associated with alternative splicing, epitope modification, or lineage switching (71, 72).

Collectively, these findings indicate that successful CAR-T therapy in solid tumors requires not only effective target selection but also strategies capable of overcoming tumor heterogeneity, TME-mediated suppression, T-cell exhaustion, and adaptive resistance. These challenges have driven the development of next-generation engineering approaches, which are discussed in the following section.

## 5 Engineering strategies to overcome biological barriers in solid tumors

Key evidence supporting these engineering approaches is summarized in **Table 2**. The major barriers limiting CAR-T-cell therapy in solid tumors—including insufficient tumor specificity, antigen heterogeneity, T-cell exhaustion, immunosuppressive tumor microenvironments (TMEs), and adaptive immune escape—have driven the development of next-generation engineering strategies. Rather than simply enhancing cytotoxicity, recent approaches aim to improve tumor selectivity, maintain T-cell fitness, broaden tumor recognition, and remodel the TME (**Table 2**) (19, 73, 74).

### 5.1 Improving tumor selectivity

Because most solid-tumor antigens are also expressed in normal tissues, improving target specificity remains a major priority. Affinity-tuned CARs preferentially recognize tumor cells expressing high antigen densities while sparing normal tissues with physiological antigen expression (83). More sophisticated approaches employ synthetic biology platforms such as SynNotch circuits and logic-gated CARs, which require sequential or combinatorial antigen recognition before T-cell activation occurs (22, 84–86). These systems have shown improved discrimination between malignant and normal tissues and may expand the range of clinically targetable antigens.

### 5.2 Preserving CAR-T-cell fitness and preventing exhaustion

Persistent antigen stimulation and chronic inhibitory signaling frequently induce T-cell exhaustion, resulting in impaired proliferation and cytotoxicity (64–66). Recent studies have identified the NR4A transcription factor family as a central regulator of exhaustion-associated programs, and genetic disruption of NR4A signaling significantly improves CAR-T-cell persistence and tumor control in preclinical models (67, 68). Additional strategies include PD-1 disruption, cytokine-support modules such as IL-15, and optimization of memory-associated T-cell phenotypes (1, 87). Together, these approaches aim to sustain long-term functionality within hostile tumor environments. Recent perspectives have further challenged the traditional assumption that long-term persistence is always required for optimal efficacy in solid tumors. Albelda proposed that repeated administration of short-lived effector CAR-T cells may, in some settings, be preferable to highly persistent central-memory-dominated products by reducing exhaustion and maintaining a functional effector pool during prolonged tumor exposure (88).

### 5.3 Expanding target coverage and preventing antigen escape

Tumor heterogeneity and antigen loss frequently lead to treatment failure following single-antigen targeting (21, 89). To address this challenge, dual-target CARs, tandem CARs (TanCARs), bicistronic CARs, and trivalent CAR constructs have been developed to broaden tumor recognition and reduce the emergence of resistant clones (79, 80, 89–91). For example, simultaneous targeting of HER2, IL13Rα2, and EphA2 improved recognition of heterogeneous glioblastoma populations and enhanced antitumor activity compared with monospecific CAR-T cells (79). Increasing evidence suggests that multi-antigen targeting will be required for durable tumor control in most solid malignancies.

### 5.4 Remodeling the tumor microenvironment

The immunosuppressive TME remains one of the principal obstacles to effective CAR-T-cell therapy (92–94). To overcome this barrier, armored CAR-T cells have been engineered to secrete cytokines such as IL-12, IL-15, or IL-18, thereby enhancing immune-cell recruitment and improving CAR-T-cell persistence (93, 95, 96). Other strategies focus on targeting suppressive stromal or immune-cell populations, including tumor-associated macrophages (TAMs), or blocking inhibitory pathways such as TGF-β signaling (81, 95–101). These approaches seek not only to enhance CAR-T-cell function but also to convert the TME from an immunosuppressive to an immune-supportive state.

### 5.5 Combination strategies and clinical translation

Although individual engineering approaches have shown promise, accumulating evidence suggests that no single modification is sufficient to overcome the multiple barriers present in solid tumors. Consequently, combination strategies are increasingly being explored. Immune checkpoint inhibitors can restore CAR-T-cell activity and partially reverse exhaustion (102–104), while chemotherapy and radiotherapy may improve tumor sensitivity by reducing immunosuppression and enhancing antigen presentation (21, 105, 106). Emerging clinical data indicate that integrating advanced CAR designs with complementary therapeutic modalities may offer the greatest potential for achieving durable responses in solid tumors.

Overall, the field has evolved from optimizing CAR structure alone toward developing integrated therapeutic platforms that simultaneously address antigen specificity, T-cell dysfunction, tumor heterogeneity, and microenvironmental suppression. Future progress will likely depend on rational combinations of these approaches together with improved patient selection and biomarker-guided treatment strategies.

### 5.6 Which strategies have advanced furthest clinically?

Current clinical evidence suggests that CLDN18.2-targeted CAR-T cells, mesothelin-directed CAR-T cells, and multi-target CAR platforms represent the most advanced approaches in solid tumors. Among them, CLDN18.2 CAR-T therapy has produced some of the most encouraging early clinical results in gastric and gastroesophageal cancers, largely due to the restricted expression pattern of CLDN18.2 and its favorable therapeutic window. In contrast, many armored CAR and gene-edited CAR platforms remain predominantly at the preclinical or early phase I stage. These observations indicate that target selection remains as important as CAR engineering in determining clinical success.

## 6 Future perspectives

Despite substantial advances in CAR-T-cell engineering, durable clinical responses in solid tumors remain uncommon. Future progress will likely depend less on individual technological innovations and more on the integration of complementary strategies that simultaneously address antigen specificity, T-cell dysfunction, tumor heterogeneity, and microenvironmental suppression.

### 6.1 Precision target selection and personalized therapy

Target selection remains the fundamental determinant of CAR-T-cell efficacy and safety in solid tumors. Emerging technologies, including single-cell sequencing, spatial transcriptomics, proteomics, and immunopeptidomics, are providing unprecedented insights into tumor antigen landscapes and may facilitate the identification of more selective and biologically relevant targets (22, 107). Future approaches are expected to prioritize antigens that are highly expressed, clonally conserved, and functionally important for tumor survival, thereby reducing the risk of antigen escape and improving therapeutic durability.

### 6.2 Programmable and exhaustion-resistant CAR-T cells

Recent advances in synthetic biology and genome engineering have enabled the development of increasingly sophisticated CAR-T platforms. Logic-gated CARs, SynNotch circuits, and cytokine-armored CAR-T cells offer the potential to improve tumor selectivity while simultaneously enhancing activity within immunosuppressive microenvironments (35, 36, 108, 109). In parallel, strategies targeting exhaustion-associated pathways, including NR4A-mediated transcriptional programs and metabolic reprogramming, may improve long-term persistence and functional fitness (67, 68). These approaches are expected to play central roles in next-generation CAR-T-cell design.

While central-memory and stem-cell memory T cells remain the preferred source for most current CAR-T products because of their superior expansion capacity and long-term persistence, it remains uncertain whether persistence alone is the dominant determinant of efficacy in solid tumors. As highlighted by Albelda and colleagues (88), the highly suppressive and physically restrictive tumor microenvironment may favor alternative approaches, including repeated administration of short-lived effector CAR-T cells capable of delivering rapid cytotoxic activity before exhaustion develops. Although definitive comparative data remain limited, this perspective challenges a long-standing assumption derived largely from hematological malignancies. Future studies directly comparing memory-enriched and effector-enriched CAR-T products in solid tumors will be important for defining the optimal balance between persistence, immediate effector function, and resistance to microenvironmental suppression.

### 6.3 Beyond autologous CAR-T cells

The complexity and cost of autologous manufacturing remain major barriers to widespread clinical implementation. Genome-editing technologies have accelerated the development of allogeneic “off-the-shelf” CAR-T products, while alternative cellular platforms such as CAR-NK cells, CAR-macrophages, and iPSC-derived immune cells are rapidly emerging (22, 68, 110). Although most remain at early stages of clinical development, these platforms may ultimately improve accessibility, scalability, and safety.

Emerging *in vivo* CAR-T generation strategies using lipid nanoparticles, viral vectors, and gene-delivery systems have attracted substantial interest because they may eliminate complex ex vivo manufacturing procedures. Compared with conventional autologous manufacturing, these approaches have the potential to improve treatment accessibility and scalability. However, their application to solid tumors remains at an early stage of development. Major challenges include achieving efficient and selective *in vivo* transduction of T cells, minimizing off-target gene delivery, ensuring controlled CAR expression, and overcoming the immunosuppressive tumor microenvironment. Therefore, while *in vivo* CAR-T generation represents a promising future direction, substantial preclinical and clinical validation will be required before its feasibility and therapeutic benefit in solid tumors can be fully established.

### 6.4 Toward ecosystem-oriented immunotherapy

Increasing evidence suggests that successful treatment of solid tumors will require remodeling the entire tumor ecosystem rather than targeting malignant cells alone. Future strategies will likely combine CAR-T-cell therapy with immune checkpoint blockade, radiotherapy, targeted therapies, and approaches that modify stromal and myeloid-cell populations (22, 108, 111). Such integrated approaches may enhance endogenous antitumor immunity and provide more durable clinical responses.

Overall, the future of CAR-T-cell therapy in solid tumors is likely to be driven by the convergence of precision target discovery, programmable cell engineering, improved cellular fitness, and rational combination therapies. Rather than relying on a single breakthrough technology, meaningful clinical progress will probably emerge from integrated platforms capable of simultaneously overcoming multiple biological barriers.

### 6.5 Which approaches are most likely to succeed clinically?

A central theme emerging from this review is that successful CAR-T therapy for solid tumors will likely require simultaneous optimization of target selection, cellular fitness, and tumor-microenvironment remodeling rather than reliance on any single engineering strategy. Among the numerous approaches currently under investigation, multi-antigen targeting, armored CAR-T cells, and rational combination therapies incorporating immune checkpoint blockade appear to have the strongest translational potential.

Among currently investigated targets, CLDN18.2, mesothelin, and HER2 have generated the largest body of early clinical evidence. In particular, CLDN18.2-targeted CAR-T therapy has demonstrated some of the most encouraging clinical activity in gastrointestinal malignancies, largely owing to its relatively restricted expression pattern and favorable therapeutic window. Mesothelin and HER2 remain attractive targets because of their broad expression across multiple solid tumors, although safety concerns and antigen heterogeneity continue to limit their clinical impact.

In contrast, many next-generation platforms, including logic-gated CARs, SynNotch circuits, and *in vivo*-generated CAR-T cells, remain largely supported by preclinical evidence. While these technologies offer innovative solutions to long-standing barriers such as off-tumor toxicity and antigen heterogeneity, their clinical feasibility and scalability remain to be established.

Over the next five years, meaningful progress is likely to arise from incremental improvements in target selection, persistence, and combination strategies rather than from any single breakthrough technology. Based on current clinical and preclinical evidence, the combination of optimized target selection (such as tumor-restricted antigens exemplified by CLDN18.2), exhaustion-resistant CAR-T-cell engineering, and approaches that remodel the immunosuppressive tumor microenvironment appears most likely to achieve durable clinical benefit in patients with solid tumors.

## 7 Conclusion

CAR-T-cell therapy has transformed the treatment landscape of hematological malignancies, yet translating this success to solid tumors remains a major challenge. As discussed in this review, the limited efficacy of CAR-T cells in solid tumors arises from multiple interconnected barriers, including insufficient tumor specificity, antigen heterogeneity, impaired trafficking and persistence, immunosuppressive tumor microenvironments, and adaptive immune escape. These obstacles highlight the fundamental biological differences between hematological cancers and solid malignancies and explain why no single engineering strategy has been sufficient to achieve consistent clinical success.

Recent advances in antigen discovery, synthetic biology, genome engineering, and cellular reprogramming have generated a diverse array of next-generation CAR-T platforms designed to address these challenges. Among the approaches currently under development, multi-antigen targeting, armored CAR-T cells, exhaustion-resistant designs, and rational combination therapies have shown particularly encouraging translational potential. Importantly, accumulating evidence suggests that durable clinical responses will likely require the simultaneous optimization of target selection, T-cell fitness, and tumor microenvironment remodeling rather than reliance on any single technological innovation.

Looking forward, the integration of precision antigen discovery, programmable cell engineering, and ecosystem-oriented immunotherapy is expected to drive the next phase of CAR-T-cell development. Continued progress will depend on translating promising preclinical advances into clinically feasible and scalable therapeutic platforms. Although significant challenges remain, these innovations provide a strong foundation for expanding the clinical applicability of CAR-T-cell therapy and improving outcomes for patients with solid tumors.

A central conclusion emerging from this review is that future success in solid tumors will likely depend on integrated therapeutic platforms rather than isolated engineering advances. Specifically, the convergence of optimized antigen selection, resistance to T-cell exhaustion, and active remodeling of the tumor microenvironment appears more important than further increases in CAR signaling potency alone. This conceptual shift—from enhancing CAR-T-cell cytotoxicity toward coordinating tumor ecosystem remodeling—may represent a critical step toward achieving durable clinical responses in solid malignancies.
